# Reducing Short-Wavelength Blue Light in Dry Eye Patients with Unstable Tear Film Improves Performance on Tests of Visual Acuity

**DOI:** 10.1371/journal.pone.0152936

**Published:** 2016-04-05

**Authors:** Minako Kaido, Ikuko Toda, Tomoo Oobayashi, Motoko Kawashima, Yusaku Katada, Kazuo Tsubota

**Affiliations:** 1 Department of Ophthalmology, Keio University School of Medicine, Shinanomachi, Shinjuku-ku, Tokyo, Japan; 2 Wada Eye Clinic, Houjyou, Tateyama-shi, Chiba, Japan; 3 Minamiaoyama Eye Clinic, Kitaaoyama, Minato-ku, Tokyo, Japan; Justus-Liebig-University Giessen, GERMANY

## Abstract

**Purpose:**

To investigate whether suppression of blue light can improve visual function in patients with short tear break up time (BUT) dry eye (DE).

**Methods:**

Twenty-two patients with short BUT DE (10 men, 12 women; mean age, 32.4 ± 6.4 years; age range, 23–43 years) and 18 healthy controls (10 men, 8 women; mean age, 30.1 ± 7.4 years; age range, 20–49 years) underwent functional visual acuity (VA) examinations with and without wearing eyeglasses with 50% blue light blocked lenses. The functional VA parameters were starting VA, functional VA, and visual maintenance ratio.

**Results:**

The baseline mean values (logarithm of the minimum angle of resolution, logMAR) of functional VA and the visual maintenance ratio were significantly worse in the DE patients than in the controls (*P* < 0.05), while no significant difference was observed in the baseline starting VA (*P* > 0.05). The DE patients had significant improvement in mean functional VA and visual maintenance ratio while wearing the glasses (*P* < 0.05), while there were no significant changes with and without the glasses in the control group (*P* > 0.05),

**Conclusions:**

Protecting the eyes from short-wavelength blue light may help to ameliorate visual impairment associated with tear instability in patients with DE. This finding represents a new concept, which is that the blue light exposure might be harmful to visual function in patients with short BUT DE.

## Introduction

In today's highly technological and information-oriented society, people around the world and across all age groups are spending unprecedented amounts of time using computers, tablets, and cell phones at work and at home. There has been some concern about the health effects of excessive use of cell phones by young people, and chronic use of video display terminals (VDT) has caused VDT syndrome to become a leading health issue.

VDT syndrome comprises shoulder-arm-neck, neuropsychiatric, and eye-related symptoms.[1–3] The eye-related components of VDT syndrome can include ocular fatigue, foreign body sensation, redness, photophobia, dry eye (DE), and myopia and other refractive problems. DE in VDT workers has been associated with short tear break up time (BUT),[4,5] which is diagnosed based on findings of symptomatic tear film instability without evidence of corneal epithelial damage.[6,7] Patients with short BUT DE also experience visual fluctuations and deterioration. [8–10]

VDTs produce blue light that lies in the range of wavelengths from 450 nm to 495 nm, which is extremely close to that of ultra-violet rays and produces a higher amount of energy. [11] The proliferation of electronic devices in use today has been accompanied by a dramatic increase in the extent of blue light exposure and in associated health risks. Therefore, accumulating damage by blue light may be concerned. Indeed, the damage is not only dependent on the energy, but also on the maximum exposure time. [12,13] Blue light stimulates the circadian system through melanopsin cells.[14–16] Reduction of short-wavelength light may not benefit circadian rhythms. On the contrary, disruption of the circadian rhythm has been cited in associated with an array of diseases including breast and prostate cancer, diabetes, obesity, heart disease, and depression. [17] Blue light can also present a direct hazard to the eye. [18–21] Blue light is a high-energy light, and the risks to the eye from overexposure can include formation of cataracts [18,19] and development of age-related macular degeneration,[19–21] while the cornea and conjunctival tissue are damaged by exposure to ultraviolet light.

We hypothesized that blue light might play a role in visual deterioration in patients with short BUT DE, and in the current study, we investigated whether limiting exposure to blue light would be effective for improving visual function in patients with short BUT DE.

## Methods

### Subjects

The study included 22 patients with short BUT DE (10 men and 12 women, mean age 32.4 ± 6.4 years, range 23–43 years) and 18 unaffected volunteers (10 men and 8 women, mean age 30.1 ± 7.4 years, range 20–49 years) recruited at the Cornea Subspecialty Outpatient Clinic of the Department of Ophthalmology of Keio University, Minamiaoyama Eye Clinic or the Wada Eye Clinic (S1 Dataset). Diagnosis of DE disease is based on the presence of more than two of the following three terms: (1) DE symptoms, (2) Schirmer test score ≤5 mm or BUT ≤ 5 s, (3) vital staining score ≥ 3 out of 9 points which are overall epithelial damage scores. According to the Japanese Dry Eye Diagnostic Criteria,[22] short BUT DE was diagnosed by the presence of DE symptoms and BUT ≤ 5 s with no positive fluorescein staining scores. The Schirmer test score was not pertinent to the diagnosis of short BUT DE,[23,24] since aqueous deficient DE was excluded from this study. Exclusion criteria were history of ocular trauma or ocular surgery including punctal occlusion or refractive surgery occurring within 12 months, ophthalmic diseases including abnormality of the nasolacrimal drainage apparatus except for DE, or best corrected decimal visual acuity (VA) of <0.9. When both eyes were eligible for the study, the mean values for both right and left eyes in each subject were used for the data analysis. Past or present use of contact lenses did not preclude inclusion in the study.

This research followed the tenets of the Declaration of Helsinki and written informed consent was obtained from all participants after an explanation of the nature and possible consequences of the study was provided. Ethics committee approval for the examination procedures and study protocol was obtained from the Institutional Review Board of the Minamiaoyama Eye Clinic in Tokyo, Japan.

### Tear Function and Ocular Surface Evaluation

Ophthalmic examinations that were conducted for the classification and assessment of DE and control groups included conjunctival and corneal vital staining with fluorescein and BUT tests. These examinations were undertaken at least 1 day after removal of contact lenses in contact lens users. Keratoconjunctival epithelial damage was evaluated using sodium fluorescein dyes. One microliter of a preservative-free 1% sodium fluorescein solution was separately instilled into the conjunctival sac by micropipette. Overall epithelial damage was scored on a scale of 0 to 9 points as described previously.[25] Vital staining scores of <3 points were considered negative. Tear stability was assessed by the standard BUT measurement.

### Functional Visual Acuity

A functional VA measurement system (Kowa, Japan) was used to examine the sequential change in VA over time. Creamy white cathode plates of the flat screen displayed black- printed Landolt optotypes is projecting white LED, which shows emission spectrum of 455 nm at peak, from the back side of a screen. The Landolt optotypes are presented on the monitor of the equipment, and their sizes change depending on the correctness of the responses. In brief, the optotypes are displayed automatically, starting with smaller ones. When the response is correct, even smaller optotypes are presented. If the responses are incorrect, larger optotypes are presented automatically. [26]

The measurements recorded were starting VA, functional VA, and the visual maintenance ratio.[27] Starting VA was defined as the standard best-corrected VA as measured by the functional VA measurement system. Functional VA was defined as the mean value of time-wise changes in VA during the examination. The visual maintenance ratio was defined as the functional VA / the baseline VA. [28]

Functional VA was measured during a 60-s period under daily vision correction, without topical anesthesia. Subjects were allowed to blink naturally during the measurement period as they delineated the orientation of automatically presented Landolt rings by manipulation of a joystick. Functional VA testing was carried out after tear function testing.

**Fig 1:**
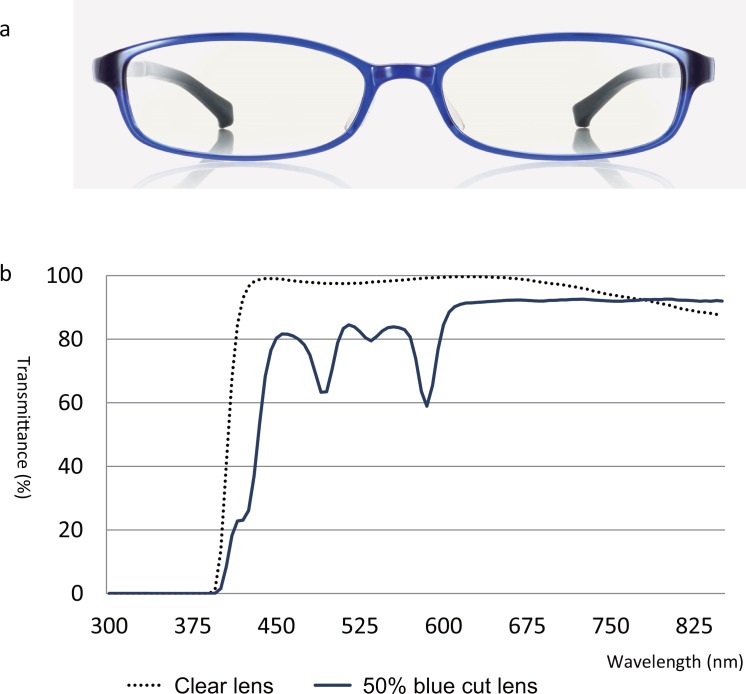
Spectacles with 50% blue light blocked lenses and spectral transmittance curve. a: Spectacles with 50% blue light blocked lenses. b: Spectral transmittance curve.

The functional VA examination was conducted first without blue light blocked lenses and secondly while wearing the spectacles with 50% blue light blocked lenses (JINS Co., Ltd, Tokyo, Japan) for 15 min, by a specialized orthoptist at each clinic, Fig 1 shows the spectacles with 50% blue light blocked lenses and the light spectral transmittance curve.

The room temperature was maintained at 23–25°C during examinations, with 60–65 humidity.

### Statistical Analysis

Student’s *t*-test was performed to compare the baseline tear function and functional VA parameters in the two groups. Functional VA parameters with and without blue light blocked glasses were compared using a paired t-test. SPSS software version 17.0J for Windows (SPSS Inc., Chicago, Illinois, USA) was used for statistical analysis. A *P*-value of < 0.05 was considered statistically significant.

## Results

### Tear Function Assessment

The tear function profiles for DE and control groups are shown in Table 1.

**Table 1:** Characteristics.

	Dry eye group N = 22	Control group N = 18
Age (years)	32.4 ± 6.4	30.1 ± 7.54
Tear function assessment		
BUT (s)	3.7 ± 2.5*	8.9 ± 1.5
Vital staining score (pts)	0.09 ± 0.3	0.4 ± 1.9
Functional Visual Acuity (VA) parameters		
Starting VA	-0.11 ± 0.07*	-0.13 ± 0.07
Functional VA	0.19 ± 0.44*	-0.08 ± 0.08
Visual maintenance ratio	0.96 ± 0.03	0.98 ± 0.02

Student’s *t*-test

*p < 0.05

BUT: break up time of tear film.

### Functional Visual Acuity

**Fig 2:**
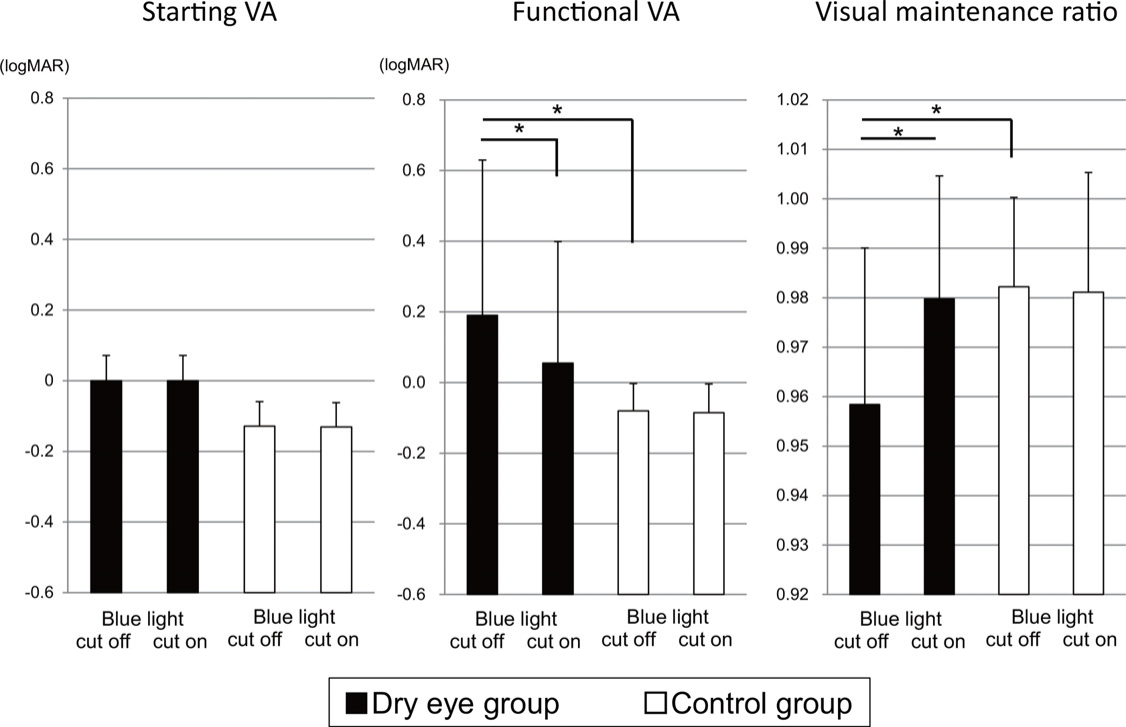
Functional visual acuity (VA) parameters with and without blue light blocked glasses.

Baseline functional VA parameters for the DE and control groups are shown in Table 1. The baseline mean values (logarithm of the minimum angle of resolution, logMAR) of functional VA and the visual maintenance ratio were significantly worse in the DE patients than in the controls (*P* < 0.05), while no significant difference was observed in the baseline starting VA (*P* > 0.05). Fig 2 shows the results of functional VA testing without and with the blue light blocked glasses. The DE patients had significant improvement in mean functional VA and visual maintenance ratio while wearing the glasses (*P* < 0.05), while there were no significant changes with and without the glasses in the control group (*P* > 0.05).

## Discussion

We compared the visual function with and without 50% blue light blocked glasses between individuals with short BUT DE and unaffected controls, and the mean baseline functional VA (i.e., without the glasses) was significantly lower in the DE group, as we have previously reported.[26] At the same time, we also found the DE group had a significantly worse mean starting VA than the control group. Functional VA may be a more important indicator of visual impairment in patients with short BUT DE than the starting VA, as is reflected in the finding that the visual maintenance ratio in the DE group tended to be lower than in the control group.

With regards to the effect of blue light blocked glasses on visual function in individuals with and without tear stability, we found the visual maintenance ratio was significantly improved by wearing the glasses only in the DE group, while there was no effect in the non-DE subjects. The visual maintenance ratio is an indicator of the ability to maintain the best VA, and it is suitable as a measure to compare visual function between groups with different baseline VA values (i.e., starting VAs). Thus, our results suggest that visual function was improved with wearing the blue light blocked glasses only in the DE group. Blue light blocked lenses are tinted yellow to reduce the amount of blue light and shade the vision. In general, reduced light reduces visibility. However, while wearing the glasses the control group’s sight did not worsen. Furthermore, interestingly, visual function was even improved in patients with short BUT DE when looking though the dark yellow lenses. We infer that blue light protection was effective in patients with short BUT DE because of the contribution of light scattering to visual function associated with tear stability.

**Fig 3:**
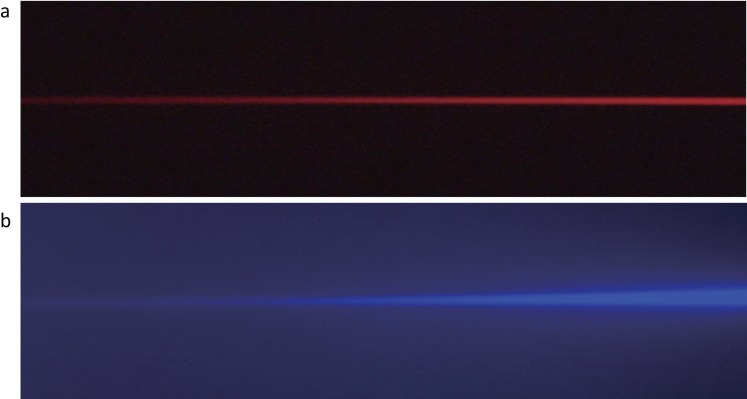
Rayleigh scattering phenomenon. a. The red light passes through the skim milk in a glass. b. The blue light scatters and little of it passes through the skim milk in a glass.

**Fig 4:**
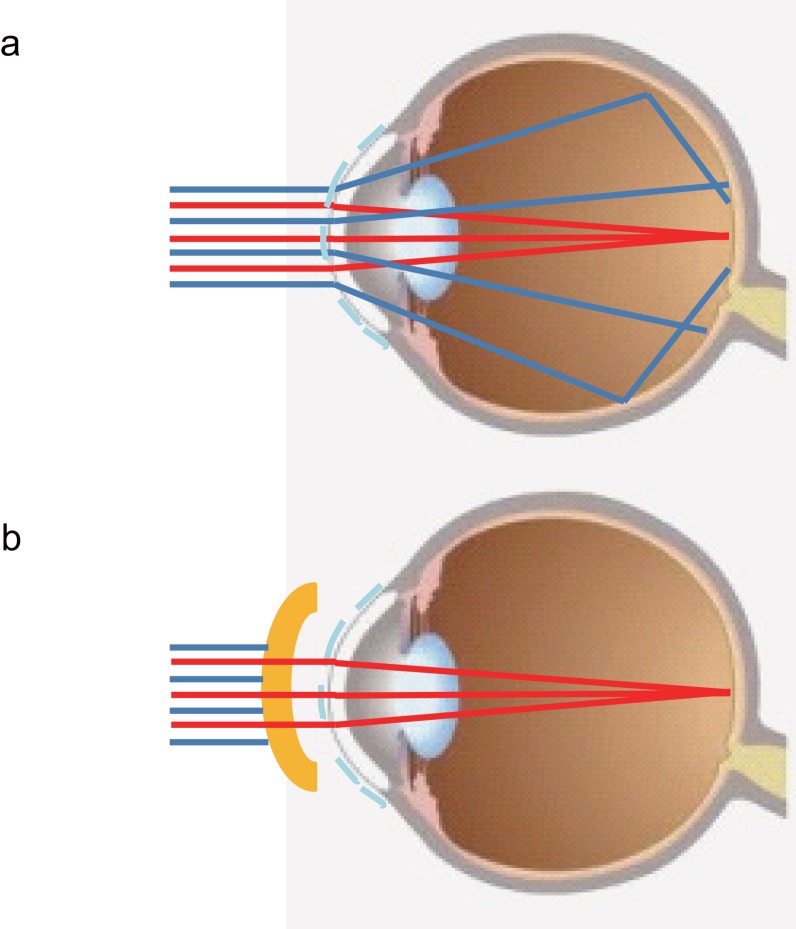
Mechanism of the effect of the blue light blocked lens on visual function. Red and blue lines represent red and blue light, respectively. a. Unstable tear film without blue light blocked lens. The blue light is scattering. b. Unstable tear film with blue light blocked lens. The lens is blocking the blue light.

The shorter wavelengths of visible white light, such as blue light, are more strongly scattered than the longer wavelengths (Fig 3). Scattering can occur when light passes through a cloud of small particles and through liquids. As is known, optical quality is deteriorated in cases of DE with decreased tear stability.[9,10] Kobashi et al.[29] evaluated the optical quality using an objective scatter index with an optical quality analysis system (OQAS, Visiometrics, Terrassa, Spain) and proved that tear film dynamics have an impact on intraocular scattering. [29] In brief, they showed that intraocular scattering degrades with time after a blink in short BUT eyes, while there is no impact on the scattering, even after blink suppression, in normal eyes. They suggested that the increase of intraocular scattering could have a considerable impact on the deterioration of visual performance in eyes with decreased tear stability. In the present study, we focused specifically on the impact of the blue wavelength on visual parameters related to tear instability and found that visual function was indeed improved with blue light protection in DE patients. The irregular tear film in cases of short BUT DE might lend to more light scattering during blue light exposure. By limiting blue light exposure, scattering that is incidentally developed by the unstable tear film might be averted, allowing improvement of optical quality and, thus, visual function. Fig 4 shows the mechanism of the blue light blocked lens effect on visual function. Scattering may also aggravate other DE symptoms, including photophobia and eye fatigue, leading to headaches and physical and mental fatigue caused by long exposure to VDTs or other electronic screen-based devices. Finally, protecting eyes from the harmful effects of blue light may be effective not only for maintaining visual acuity and reducing DE symptoms, but also for regulating the circadian rhythm.

There are a few limitations to this study. A major limitation is that we did not account for the lens characteristics in the blue cut glasses, which may impact on the resulting images (e.g., color, subtle gradations in tone, impact-enhancing contrast, and sharpness-enhancing resolution). We should have tested lenses that cut the same amount of light without cutting blue wavelength in the comparison in order to avoid effects on visual function due to the lens characteristics. The other limitation is that we did not assess light scattering. There are two instruments available in Japan for measuring intraocular light scattering, the OQAS and the C-Quant Straylight Meter (Oculus GmbH, Wetzlar, Germany). The former is an objective examination, which is preferable for assessing light scattering corresponding to tear dynamics. Unfortunately, the light source of the OQAS does not include short-wavelength blue, and so blue light effects cannot be assessed. The latter is a subjective examination based on the compensation-comparison method, which may not reflect actual tear dynamics because it is performed under blinking.

In conclusion, we demonstrated a possible relationship between tear stability and visual function in blue light exposure. Limiting exposure to short wavelength blue light may help to reduce visual impairment in patients with short BUT DE. This study suggests a new concept, that the blue light exposure might be harmful to visual function in patients with DE with unstable tear film. We hope to conduct a further study to examine the relationship between blue light scattering and tear dynamics.

## Supporting Information

S1 Dataset Raw Data. (XLSX)
